# Vertebral Artery Compression Syndrome

**DOI:** 10.3389/fneur.2019.01075

**Published:** 2019-10-15

**Authors:** Qi Li, Peng Xie, Wen-Song Yang, Bernard Yan, Stephen Davis, Louis R. Caplan

**Affiliations:** ^1^Department of Neurology, The First Affiliated Hospital of Chongqing Medical University, Chongqing, China; ^2^Department of Neurology, The Royal Melbourne Hospital, Melbourne, VIC, Australia; ^3^Department of Neurology, Beth Israel Deaconess Medical Center, Harvard Medical School, Boston, MA, United States

**Keywords:** vertebrobasilar dolichoectasia, intracranial arterial dolichoectasia, stroke, vertebral artery, compression, imaging

## Abstract

**Objective:** We aim to propose the term “vertebral artery compression syndrome” to describe a group of patients with a variety of clinical symptoms caused by vertebral artery compression of the medulla or spinal cord.

**Methods:** We conducted the prospective case study in a university teaching hospital. Eleven patients who fulfilled the diagnostic criteria of vertebral artery compression syndrome and 22 age- and sex- matched controls were recruited. Clinical presentation and radiological findings of patients with vertebral artery compression syndrome were assessed and recorded. The basilar artery diameter was measured at the midpons level on T2 weighted MR images and compared between both groups.

**Results:** Medullary compression was observed in 10 of 11 patients. The most common clinical presentation is dizziness, vertigo, imbalance, or ataxia followed by limb weakness. Cervical spinal cord compression was observed in one patient who presented with neck pain and left leg weakness. The mean basilar artery diameter was similar between patients and controls (3.95 ± 0.41 vs. 3.81 ± 0.43 mm).

**Conclusions:** Vertebral artery compression of medulla and spinal cord may cause various clinical symptoms. Future studies are needed to further clarify the prevalence, natural history and treatment of this condition.

## Introduction

Vertebrobasilar dolichoectasia, or intracranial arterial dolichoectasia, is an uncommon neurovascular disorder characterized by elongation and enlargement of the vertebrobasilar arteries (1–3). This well-established vascular anomaly has been associated with subsequent strokes, microembolization, brainstem, and cranial nerve compression (3–7). In previous studies, dolichoectasic basilar artery compression of the pons and trigeminal nerve entry zone have been widely recognized (3, 8, 9), and medullary compression has been described (10).

Since many patients with vertebrobasilar dolichoectasia remains asymptomatic and the term “vertebrobasilar dolichoectasia” is more likely a description of anatomical abnormality or variation rather than a clinically significant syndrome. Recently, we have observed a variety of neurological symptoms that share a common vascular pathology-compression of the medulla or spinal cord by a vertebral artery. The clinical presentations of these patients make up a wide spectrum of symptoms including dizziness, vertigo, ataxia, dysarthria, dysphagia, progressive or acute paralysis, hemisensory loss, and cervical myelopathy. The clinical features are non-specific and difficult to diagnose. For these reasons, it is worthwhile to propose a new name for this interesting but uncommon disorder. We propose the term “vertebral artery compression syndrome” (VACS) for this condition. This syndrome is unfamiliar to many clinicians and is under-recognized in clinical practice. We aim to investigate the clinical and radiological characteristics of patients with VACS and compare the basilar artery diameter with age- and sex- matched controls.

## Methods

We prospectively included patients that fulfill our proposed definition of VACS seen from March 2013 to November 2017 in our hospital. The definition of VACS was proposed by Dr. Qi Li and VACS was operationally defined as: Imaging evidence of compression of the medulla/upper spinal cord by vertebral artery and the compression causing corresponding symptoms. Patients with imaging evidence of vertebral artery compression of brainstem and spinal cord was screened by Dr. Qi Li. Patients were included in the study if they had imaging evidence of compression of the medulla/upper spinal cord by vertebral artery and the compression is responsible for corresponding symptoms. Patients were excluded if they had abnormal brain imaging findings that better explain the clinical symptoms. Patients with acute ischemic stroke were also excluded. A control group of 22 subjects without vertebral artery compression matched for age and sex was recruited.

The baseline demographic information, clinical signs and symptoms, and brain and vascular imaging findings were collected. The relationship of the vertebral arteries to intracranial structures was evaluated using MRI scanning. T2 weighted MR images were used to observe the relationship between the vertebral arteries and the medulla oblongata and the cervical spinal cord. The basilar artery diameter was measured at the midpons level on T2 weighted MR images. Basilar artery dolichoectasia was defined as a basilar artery diameter at the midpons >4.5 mm according to previous definitions (2, 7). The vertebral artery diameter was measured at the site of compression. Vertebral artery dominance was assessed in all patients. Vertebral artery dominance was considered if the patient had a side to side vertebral artery diameter difference ≥0.3 mm (11). A hypoplastic vertebral artery was defined as a V4 diameter of ≤2.0 mm according to previous definitions (12, 13). This study was approved by the ethics committee of The First Affiliated Hospital of Chongqing Medical University. Informed consent was obtained from all participants or their legal representatives.

## Results

A total of 11 patients (4 men and 7 women) who had the clinical and imaging characteristics of VACS and 22 age- and sex- matched controls were included in our study. The average age was 63.8 years (age range 41−82 years). The clinical and imaging characteristics of patients with vertebral artery compression syndrome are summarized in Table 1.

**Table 1:** Clinical and imaging characteristics in patients with VACS.

**Patient NO./sex/Age, year**	**Medical conditions**	**Symptoms, treatment, and outcome**	**Neurologic examination result**	**Location of medullary compression**
1/F/59	Unremarkable	Progressive left leg weakness and imbalance, no specific treatment, symptom persists	Decreased left leg muscle strength, hyperreflexia in four limbs	Left anterolateral compression of medulla by left VA
2/M/82	Coronary artery disease	Right lower limb weakness and dizziness, non-specific treatment, symptom persists	Decreased right leg muscle strength, positive Romberg test and incoordinate finger to nose test	Right anterolateral compression of medulla by right VA
3/F/52	Hypertension	Left sided weakness non-specific treatment, symptom persists	Decreased left limb muscle strength	Left anterolateral compression of medulla by left VA
4/F/51	Hypertension	Left lower limb weakness, imbalance, veering to the left non-specific treatment, symptom persists	Decreased left limb muscle strength	Left anterolateral compression of medulla by left VA
5/M/61	Diabetes	Left sided weakness and numbness non-specific treatment, symptom persists	Decreased left side muscle strength	Right anterolateral compression of medulla by right VA
6/M/41	Unremarkable	Dysphagia and imbalance non-specific treatment, symptom persists	Weak pharyngeal reflex	Left anterolateral compression of medulla by left VA
7/F/72	Hypertension diabetes	Dysarthria and ataxia, symptom persists	Incoordinate finger to nose test	Left anterolateral compression of medulla by left VA
8/F/71	Hypertension and diabetes	Dysarthria and imbalance, improved after treatment with aspirin	Incoordinate finger to nose test and heel knee sheen test	Left anterolateral compression of lower medulla by Left VA
9/F/66	Pulmonary tuberculosis	Vertigo and imbalance, improved after treatment with aspirin	Positive Romberg sign	Left anterolateral compression of medulla by left VA
10/F/73	Unremarkable	Vertigo and imbalance, improved after treatment with aspirin	Unsteady gait	Left anterolateral compression of medulla by left VA
11/M/74	Diabetes, coronary heart disease	Nape pain, left leg weakness, symptom persists after physiotherapy	Decreased left leg muscle strength	Left anterolateral compression of cervical spinal cord by left VA

*VACS, indicates vertebral artery compression syndrome; VA, vertebral artery*.

### Clinical Findings

Medullary compression was observed in 10 of 11 patients. Of the 10 patients with medullary compression, dizziness, imbalance, vertigo, or ataxia were observed in 8 patients. Five patients had limb weakness. Two patients had dysarthria. One patient had dysphagia. Cervical spinal cord compression was observed in one patient who presented with neck pain and left leg weakness. The nature of the clinical findings depended on whether the brainstem or cervical spinal cord was compressed.

### Imaging

Brain MRI was performed in all 11 patients and 22 controls. The mean basilar diameter did not differ significantly between patients and controls (3.95 ± 0.41 vs. 3.81 ± 0.43 mm). In patients with VACS, vertebral artery dominance was observed in 10 of 11 (90.9%) patients with VACS. Right vertebral artery hypoplasia was observed in 4 patients. Of the 11 patients with VACS, medullary compression was observed in 10 patients. One patient had cervical spinal cord compression.

### Representative Case Reports

**Figure 1:**
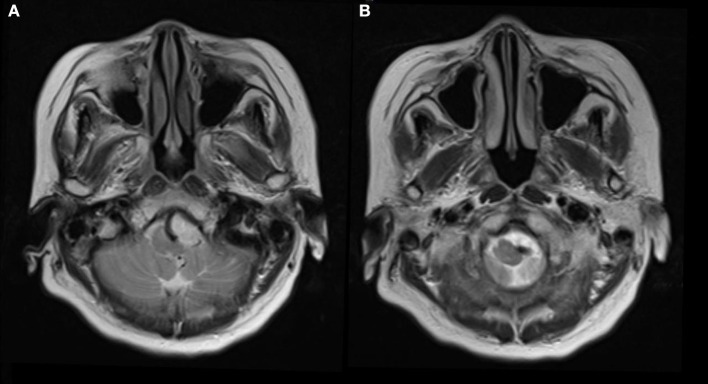
Seventy-three-year-old woman presented with vertigo and imbalance. Magnetic resonance imaging showed severe compression and indentation **(A)** of the left lower medulla. Note that the medulla was displaced to the right side **(B)** by the tortuous vertebral artery.

A 73-year-old hypertensive woman suddenly lost her balance while walking out of an elevator. She felt that the ground and adjacent objects were moving and that she was swaying. There was no tinnitus, hearing loss, or a fullness in the ear. The vertigo was not triggered by specific changes in the position of her head. Neurological examination revealed unsteady gait and was otherwise normal. MRI scan was performed and showed no acute infarcts on diffusion-weighted imaging scan. Severe indentation of the left medulla by a tortuous vertebral artery was observed on T2 weighted MR image (Figure 1).

A 59-year-old woman presented with progressive left leg weakness, spasticity and imbalance for 2 years. In the past, she was always healthy. On examination, she had spasticity in four limbs with exaggerated deep tendon reflexes and left lower extremity muscle strength was decreased. MRI of the brain revealed anterolateral compression of the left base of the medulla oblongata by a tortuous vertebral artery. The patient received physiotherapy. The symptoms persisted.

**Figure 2:**
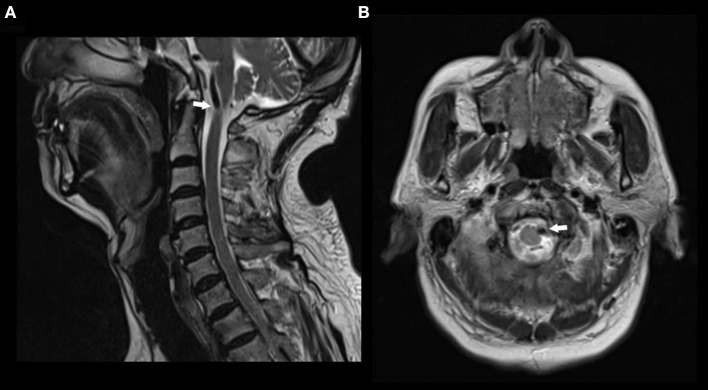
MR images in a patient with nape pain and left leg weakness. **(A)** Sagittal T2 weighted MR image showing a signal void compressing the upper cervical spinal cord at the atlas level. **(B)** Axial T2 weighted MR image showing anterolateral compression of the spinal cord by left vertebral artery.

A 74-year-old man had pain in the area of the neck and trapezius muscle and left leg weakness. He had a history of diabetes for 17 years. He was diagnosed with coronary artery disease 1 month before presentation. On neurological examination, the patient had decreased left leg muscle strength. MRI scan showed a signal void region at the level of the atlas. Axial MR image revealed left anterolateral compression of the cervical spinal cord near the cranial-spinal junction by the left vertebral artery (Figure 2).

## Discussion

In our study, we demonstrated that vascular compression of brainstem or cervical spinal cord can present with various signs and symptoms. This syndrome, which we call VCAS, is distinct from basilar artery dolichoectasia. Dolichoectasia of the basilar artery has been associated with compression of the pons, cranial nerve palsies, and even ischemic events (2, 4, 9). The most widely used diagnostic criteria for vertebrobasilar dolichoectasia were proposed by smoker et al. (7). Basilar artery diameter at the midpons >4.5 mm was considered dolichoectasic (4, 7). In our study, we are interested to observe that none of our patients with VACS had MRI evidence of basilar artery dolichoectasia. Therefore, we did not use the term vertebrobasilar dolichoectasia to describe this condition. In addition, vertebrobasilar dolichoectasia literally only describes the anatomical feature of a dilated arteriopathy. The current diagnostic criteria for vertebrobasilar dolichoectasia was based on imaging morphology but not clinical symptoms. In this report, we term this condition vertebral artery compression syndrome because all symptoms were caused by compression of medulla oblongata or cervical spinal cord with an offending vertebral artery. The diagnosis of VACS is especially challenging for clinicians since this condition is not described as an entity in the literature. We did not define the syndrome based on the anatomic characteristics of the vertebral artery i.e., the diameter or length of the vertebral artery. More importantly, recent studies show that a significant proportion of patients with dolichoectasic and dilated vertebral artery are asymptomatic and may have neurovascular contact (14). Therefore, it is very important to differentiate asymptomatic verse symptomatic vertebral artery compression of medulla or spinal cord. Based on these findings, we propose that patients should have both imaging evidence of vascular compression of the medulla oblongata or cervical spinal cord and had relevant clinical symptoms.

Vascular compression of brainstem is a little-known entity in the medical literature. Vertebral artery compression of the medulla has been described in a few case reports (10, 14–17). In 2006, Savitz et al. described nine patients with medullary compression by a tortuous vertebral artery which is the largest case series reported in the literature (10). Our study includes another ten patients with medullary compression by a vertebral artery. Among our reported patient, the most common clinical signs and symptoms were dizziness, vertigo, imbalance, and limb weakness. Dysarthria was also observed in two patients. Three out of nine patients with medullary compression by a vertebral artery reported in the 2006 Savitz et al. report had dizziness, imbalance or vertigo (10). Among our patients, 4 of 10 with medullary compression presented with dizziness, vertigo, imbalance and ataxia. Other important and common findings in patients with vertebral artery compression of medulla are hemiparesis, a finding present in five of our patients. Patients may present with acute onset of symptoms or manifest as a slowly progressive course, depending on the mechanism of injury. A total of 14 patients with vertebral artery compression of medulla were reported before 2006 (10). Among these 14 patients, 11 had hemiparesis, quadriparesis, or sensory symptoms. Eight of these 11 patients had microvascular decompression surgery and all except one had improvement of symptoms. This suggests that there is a causal relationship between vascular compression and corresponding symptoms. Hemiparesis, quadriparesis, or sensory symptoms are more commonly described in the literature than dizziness, vertigo, and imbalance. A possible explanation is these symptoms may more often prompt vascular imaging. We found that compression of the anterolateral surface of the medulla is common and may be responsible for these symptoms. The corresponding symptoms may be ipsilateral or contralateral depending on the site of medullary compression. Compression of the corticospinal tract below the pyramidal deccussation may cause ipsilateral weakness and pyramidal tract signs, whereas compression above pyramidal deccussation is responsible for contralateral symptoms.

An offending vertebral artery may cause symptoms through several potential mechanisms. Anterolateral compression of the medulla oblongata is the most common cause of VACS. The pulsatile impact of a tortuous vertebral artery on an impingement location may be responsible for patients with recurrent symptoms or transient symptoms. Ischemic injury may be another potential mechanism of injury in patients with transient symptoms. Tortuosity of the vertebral artery and compression of the brainstem may cause blood flow insufficiency in perforating branches which may lead to transient symptoms. If the impingement is severe and does not revolve, patients may have progressive symptoms.

Our report also includes a patient with cervical myelopathy due to vertebral artery compression of the rostral spinal cord. Vertebral artery compression of the upper spinal cord is an extremely rare cause of cervical myelopathy. To the best of our knowledge, a total of 15 patients with cervical myelopathy due to vertebral artery compression have been reported in the literature (18). Patients present with a variety of symptoms including nape pain, sensory disturbances, and spasticity. The spinal cord compression may be unilateral or bilateral (19).

### Treatment

The ideal methods of treatment for VACS remains unknown. Several authors have described the results of surgical treatment of the condition by microvascular decompression (MVD). The first microvascular decompression surgery for medullary compression was performed by Kim et al. in a patient presenting with progressive hemiparesis secondary to vertebral artery compression of the medulla oblongata (20). Eight patients with pyramidal tract weakness due to medullary compression by a vertebral artery were treated by MVD in the literature before 2016 (21). Patients with pyramidal weakness who had MVD surgery had some improvement of symptoms or even complete recovery after the procedure. MVD surgery has been shown to be effective in medullary compression patients who presented with dysphagia, respiratory compromise, hoarseness, and obstructive sleep apnea (10, 22, 23). Mobilization and anchoring of the vertebral artery to the spinous process or the dura has been shown to be an effective treatment option for cervical myelopathy secondary compression by anomalous vertebral artery in five cases reported in the literature (19). Although a few case reports described improvement of symptoms after microvascular decompression, Savitz et al. only noted slight improvement in the two patients they referred for surgery. We propose that irreversible damage may occur after prolonged compression and the effect of surgery on functional outcome varied widely on an individual basis. A limitation of previous reports on MVD surgery is the lack of long-term follow up in most patients. Patients with VACS should be treated on an individual basis.

Our study has several limitations. First, the sample size is relatively small. Second, decompressive surgery or advanced imaging such as diffusion tensor imaging was not performed in patients with VACS. Future studies with large number of patients and long-term follow-up are needed to further clarify the optimal treatment for VACS.

## Ethics Statement

All subjects gave written informed consent in accordance with the Declaration of Helsinki. The protocol was approved by the ethics committee of The First Affiliated Hospital of Chongqing Medical University.

## Author Contributions

QL: study concept, design, and drafting of manuscript. QL, PX, SD, and LC: revising and important intellectual content. All authors: acquisition, analysis, or interpretation of data for the work.

### Conflict of Interest

The authors declare that the research was conducted in the absence of any commercial or financial relationships that could be construed as a potential conflict of interest.
